# DISC1 knockdown impairs the tangential migration of cortical interneurons by affecting the actin cytoskeleton

**DOI:** 10.3389/fncel.2014.00190

**Published:** 2014-07-08

**Authors:** André Steinecke, Christin Gampe, Falk Nitzsche, Jürgen Bolz

**Affiliations:** Universität Jena, Institut für Allgemeine Zoologie und TierphysiologieJena, Germany

**Keywords:** DISC1, Schizophrenia, interneuron, cortical interneuron migration, cortical development

## Abstract

Disrupted-in-Schizophrenia 1 (DISC1) is a risk gene for a spectrum of major mental disorders. It has been shown to regulate radial migration as well as dendritic arborization during neurodevelopment and corticogenesis. In a previous study we demonstrated through *in vitro* experiments that DISC1 also controls the tangential migration of cortical interneurons originating from the medial ganglionic eminence (MGE). Here we first show that DISC1 is necessary for the proper tangential migration of cortical interneurons in the intact brain. Expression of EGFP under the Lhx6 promotor allowed us to analyze exclusively interneurons transfected in the MGE after *in utero* electroporation. After 3 days *in utero*, DISC1 deficient interneurons displayed prolonged leading processes and, compared to control, fewer neurons reached the cortex. Time-lapse video microscopy of cortical feeder-layers revealed a decreased migration velocity due to a reduction of soma translocations. Immunostainings indicated that DISC1 is co-localized with F-actin in the growth cone-like structure of the leading process. DISC1 knockdown reduced F-actin levels whereas the overall actin level was not altered. Moreover, DISC1 knockdown also decreased levels of phosphorylated Girdin, which cross-links F-actin, as well as the Girdin-activator pAkt. In contrast, using time-lapse video microscopy of fluorescence-tagged tubulin and EB3 in fibroblasts, we found no effects on microtubule polymerization when DISC1 was reduced. However, DISC1 affected the acetylation of microtubules in the leading processes of MGE-derived cortical interneurons. Together, our results provide a mechanism how DISC1 might contribute to interneuron migration thereby explaining the reduced number of specific classes of cortical interneurons in some DISC1 mouse models.

## Introduction

Disrupted-in-Schizophrenia 1 (DISC1) was originally discovered in a Scottish family in which a chromosomal translocation breaks this gene. More than 70% of those family members with DISC1 disruption were diagnosed as being schizophrenic, unipolar depressive or bipolar depressive (Millar et al., 2000). Many studies afterwards firmly established that DISC1 plays a major role in early brain development by regulating a number of essential neurodevelopmental events, including cell proliferation, neurite outgrowth, synapse formation and neuronal migration (for reviews see Ishizuka et al., 2006; Brandon et al., 2009; Soares et al., 2011; Narayan et al., 2013; Thomson et al., 2013). These pleiotropic actions of DISC1 are attributed to its many interaction partners, the DISC1 interactom (Camargo et al., 2007), making DISC1 a scaffold protein that impacts on many diverse brain functions.

We recently provided *in vitro* evidence that during embryonic development DISC1 is a necessary component for the correct tangential migration of cortical interneurons from the medial ganglionic eminence (MGE) to their target regions in the cortex (Steinecke et al., 2012). This is in accordance with previous studies which found a reduced number of parvalbumin-positive interneurons in a mouse line that expresses the truncated human DISC1, as in the Scottish pedigree (Pletnikov et al., 2008; Ayhan et al., 2011). In the present study we first demonstrate the importance of DISC1 during tangential migration in the intact brain. For this we performed *in utero* electroporations and used constructs that either reduce endogenous DISC1 levels or that express a truncated murine form of DISC1 in combination with a vector that was only expressed in MGE-derived neurons. We then examined how DISC1 interferes with the cellular and the molecular machinery that drives glia-independent neuronal migration. We found that DISC1, by interacting with Girdin (girders of actin filaments) and Akt (also known as protein kinase B), regulates actin polymerization. In addition, DISC1 also influences the stability of microtubules. Thus, both cytoskeletal elements that have been implicated in interneuron migration, actin filaments and microtubule, are modified by DISC1.

Our results indicate that DISC1 has an impact on the migratory behavior of interneurons during early development that might lead to changes in the number or composition of interneurons in the cortex. Together, this work is supporting the hypothesis that subtle perturbations in the developing brain may increase the risk for neuropsychiatric diseases later in life (Murray and Lewis, 1987; Weinberger, 1987; Cannon et al., 2002; Owen et al., 2011).

## Materials and methods

### Mice

Animals used were timed pregnant C57BL/6 mice. The day of insemination was considered as embryonic day (E) 1. Mice were killed using peritoneal injection of 10% chloral hydrate. All animal procedures were performed in agreement with the institutional regulations of the University of Jena.

### Plasmids

Vectors expressing miRNA for DISC1 knockdown and control transfection were described previously (Steinecke et al., 2012). EmGFP has been removed for cotransfection. Additional vectors used: pLhx6-IRES-GFP (gift from Dr. Anderson), pCAX 1-597 (short DISC1, gift from Dr. Sawa), pEB3-GFP (gift from Dr. Galjart), pTubulin-GFP (coding sequence for α-tubulin cloned into pEGFP-C1 between XhoI and BamHI), pActin-RFP (coding sequence for β-actin cloned into pmRFP-C1 between XhoI and BamHI).

### *In utero* electroporation

Timed pregnant mice (E13.5) were treated with 4 mg/kg Carprofen for 20 min before deeply anesthetizing with a mixture of fentanyl (0.05 mg/kg), midazolam (5 mg/kg), and metedomidine (0.5 mg/kg). After the uterine horns were exposed various constructs together with pLhx6-IRES-GFP in a 4:1 ratio (2 μg/μl DNA at all) were injected into the lateral ventricles of the embryos and electroporation (5 pulses 40 V, 100 ms duration) was carried out with a forceps electrode connected to a BTX ECM 830 (Harvard Apparatus). After 3 days *in utero* embryonic brains were fixed in 4% paraformaldehyde and vibratome sections (150 μm) were immunostained against GFP (A6455, Invitrogen, 1:1000).

### *Ex utero* electroporation

Brain hemispheres from E14.5 embryos were dissected in ice-cold sterile Krebs buffer [126 mM NaCl, 2.5 mM KCl, 1.2 mM NaH_2_PO_4_, 1.2 mM MgCl, 2.1 mM CaCl_2_, 25 mM NaHCO_3_ and 11 mM glucose]. *Ex utero* electroporation was performed as described previously (Yozu et al., 2005). In brief, miRNA solution was pressure injected into the ventricular zone of the MGE, followed by electroporation of 2 pulses a 100 ms duration and 100 V using a BTX ECM 830 (Harvard Apparatus).

### Primary cell culture and immunostaining

After *ex utero* electroporation MGE were dissected and collected in ice-cold Hank's balanced salt solution (HBSS) supplemented with 0.65% glucose. After incubation with 0.025% trypsin in HBSS for 17 min at 37°C, tissue was dissociated into single cells by trituration and filtered through a nylon gauze to remove cell aggregates. Neurons were cultured in Dulbecco's Modified Eagle Medium (DMEM) [supplemented with 10% fetal bovine serum (FBS), 100 U/ml penicillin, 100 μg/ml streptomycin, and 0.4 mM l-glutamine] at 37°C and 5% CO_2_.

For immunocytochemistry cells were incubated on coated cover slips (19.5 μg/ml Laminin 5 μg/ml poly-l-lysin) and fixed after 2 div in 4% PFA for 30 min and washed in 0.2% TritonX-100 in PBS, blocked in 10% serum, 5% BSA, and 0.2% TritonX-100 in PBS for 1 h followed by the incubation of the primary antibodies for 1 h or overnight. After washing cells were incubated for 1 h with the secondary antibody. After washing, nuclei were stained with 4′,6-Diamidin-2-phenylindol (DAPI). Following antibodies have been used: anti-DISC1 [SantaCruz, DISC-1(N-16): sc-47990, 1:50]; anti-Actin (Hybridoma Bank, 1:50); anti-acTubulin (gift from Dr. Kessels, 1:400); anti-Akt/PKB[pS^473^] (Invitrogen, 1:100); anti-Girdin (S1416 phos) (IBL, 1:50). For staining of F-actin cells were incubated in phalloidin (Biotium, 1:100).

For cells stained against F-actin and actin an Axiovert-S100, 40× objective NA: 0.45 Plan Neofluar in combination with a Spot camera was used. Cells stained against DISC1, pGirdin and pAkt were scanned using a LSM510 (60× objective NA: 1.4 Plan Apochromat; argon laser with 488 nm excitation, bandpass filter 500–550 nm emission; HeNe laser with 543 nm axcitation, longpass filter 560 nm). Cells stained against acTubulin were scanned using a LSM510 (10× objective NA:1.2 C-Apochromat; argon laser with 488 nm excitation, bandpass filter 500–550 nm; HeNe laser 543 nm excitation, longpass filter 560 nm).

### Cortical feeder-layer

Feeder-layers were prepared as described previously (Bortone and Polleux, 2009). In brief, cortex cells of E14.5 embryos were cultured on coated cover slips and *ex utero* electroporated cells of the MGE were placed on top. After incubation at 37°C and 5% CO_2_ for 36 h time lapse analysis was started using a ZEISS LSM510 (20× objective NA: 0.75 Zeiss Plan Apochromat; argon laser with 488 nm excitation, bandpass filter 500–550 nm emission) in combination with an incubation chamber (37°C and 5% CO_2_).

### Outgrowth assay

For interneuron migration in a 3D substrate of plasma MGE of E14.5 embryos were dissected in MZ medium (0.4 g methyl cellulose, 5% FBS, 1% penicillin/streptomycin, 1% L-glutamine, 0.1% glucose) and cut in 200 × 200 μm pieces using a tissue shopper. The MGE explants were precultured at 37°C and 5% CO_2_ for 1 h, embedded in chicken plasma which was cross-linked with thrombine and cultured in MZ medium for 2 div before fixation with 4% paraformaldehyde. For staining of F-actin cells were incubated in phalloidin (Biotium, 1:100). For immunostaining following antibodies have been used: anti-DISC1 (SantaCruz, 1:50); anti-Tubulin (Hybridoma Bank, 1:200).

### Fibroblast cell culture

NIH3T3 fibroblasts were grown in DMEM-F12 with 10% FBS, 5% Penicillin/Streptomycin (P/S) under standard cell culture conditions, transfected with various constructs using Lipofectamine2000 (Invitrogen) according to the manufacturer's protocol and incubated for 48 h. For time lapse analysis cells were scanned using a ZEISS LSM510 (Tubulin-GFP: 40× objective NA: 1.3 Plan Neofluar, EB3-GFP: 63× objective NA: 1.4 Plan Apochromat; argon laser with 488 nm excitation, longpass filter 505 nm emission) in combination with an incubation chamber (37°C and 5% CO_2_).

### Cell analysis and statistics

Transfected cells were scanned using a Zeiss LSM510. Anaylsis of feeder-layer experiments was performed using the ZEN2011-LE software (ZEISS). Analysis of interneuron morphology as well as EB3 in fibroblasts was performed using ZEN2009 software (ZEISS). Fluorescence intensities were analyzed using ImageJ. At least three independent experiments were performed and analyzed. Significance was calculated using Student's *t*-test (Excel). For analysis of migration defects after *in utero* electroporation One-Way ANOVA [*F*_(2.99)_ = 33.826, *p* < 0.001] and *post-hoc* test (*p*-value indicated in the text) were performed using SPSS.

## Results

### Decreased tangential migration of DISC1 deficient cortical interneurons *in vivo*

Our initial hypothesis of compromised interneuron migration after DISC1 knockdown was based on functional *in vitro* assays (Steinecke et al., 2012). Therefore, in the present study, our first goal was to examine the migration pattern of DISC1 deficient interneurons in the intact brain. For this, one experimental strategy would be to electroporate different constructs that suppress DISC1 specifically in the MGE of embryonic brains *in utero*. It has been reported that the ganglionic eminences can be selectively electroporated with ventrolateral placement of the positive electrode (Borrell et al., 2005). However, we and others could not exclusively target the MGE with this technique, instead transfected cells were found scattered or in clusters throughout the basal telencephalon and at the corticostriatal junction (Bai et al., 2008; Steinecke et al., 2012). To overcome this problem, Brown et al. (2011) used transgenic mice that express the TVA receptor selectively in the VZ of the MGE and POA and then performed *in utero* intraventricular injections of RCAS retroviruses to target MGE and POA neurons.

**Figure 1:**
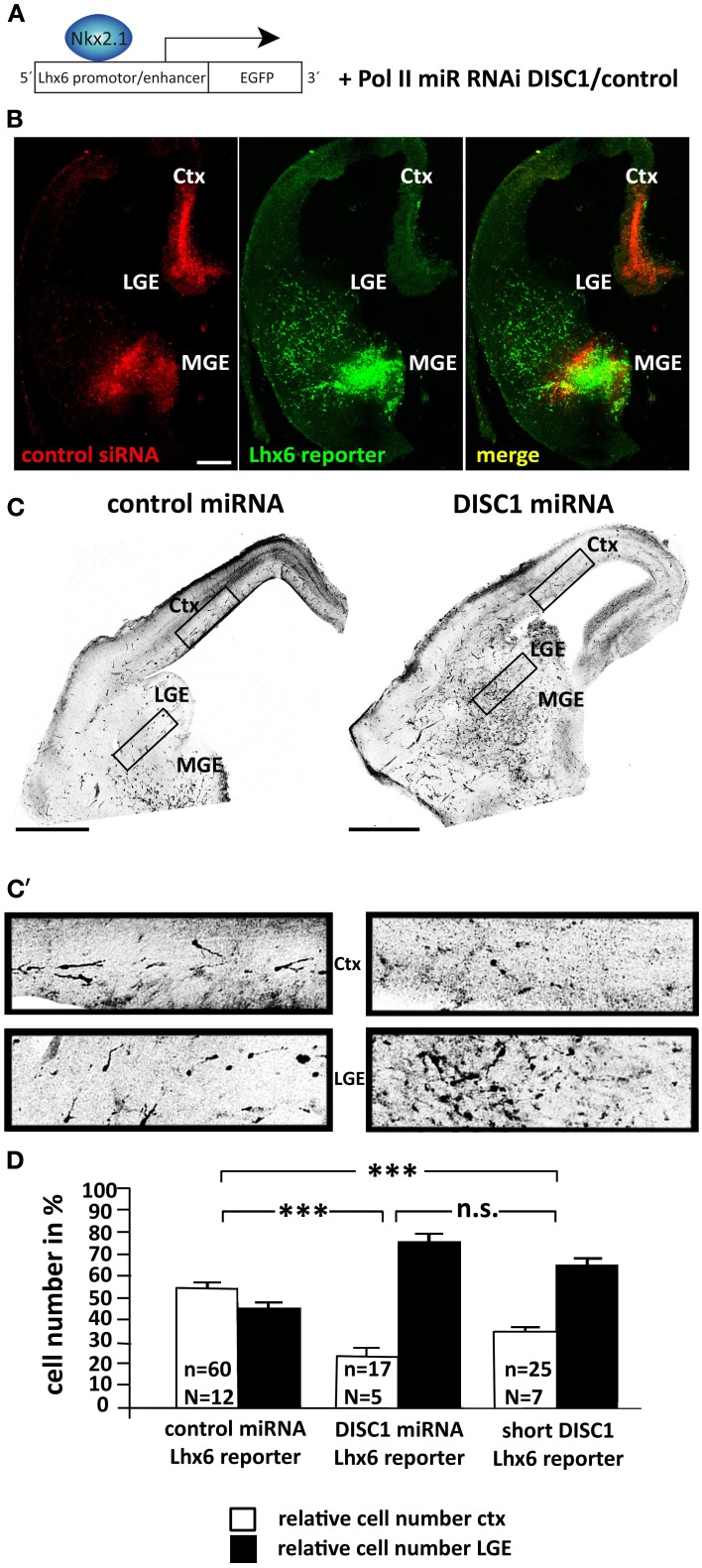
**DISC1 is necessary for the correct tangential migration of interneurons *in vivo*. (A)** Schematic of the electroporation strategy. The expression of the reporter construct is controlled by the Lhx6 promotor and labels MGE-derived interneurons. The control and DISC1 miRNA constructs were co-electroporated with the reporter vector in a 4:1 ratio. **(B)** Photo- micrographs of slice cultures that were *ex utero* electroporated with the Lhx6 reporter vector that was co-injected with Alexa555 labeled control siRNA in the cortex as well as the MGE. The reporter is only activated in the MGE, but not the cortex. Scale bar: 200 μm. **(C)** Inverted fluorescent micrographs of brain slices after *in utero* electroporation with control or DISC1 miRNA constructs as well as the Lhx6 reporter vector. Scale bars: 500 μm. **(C′)** Higher magnified parts of cortex and LGE demonstrate a different cell distribution after expression of control or DISC1 miRNA. **(D)** Quantification of cells that migrated to the LGE and the cortex resulted in a decreased number of interneurons that reached the cortex in brains expressing DISC1 miRNA or short DISC1 compared to control transfected brains. Student's *t*-test: n.s. *p* ≥ 0.05, ^***^p < 0.001. Error bars: s.e.m. “n” reflects the number of slices and “N” the number of brains.

We developed a different technique to target cortical interneurons in the embryonic brain. For this we used the vector pLhx6-EGFP as reporter construct which contains the lim-homeobox gene 6 (Lhx6) promoter. With this vector, EGFP labeling should occur only in those transfected cells in which Lhx6 expression is stimulated by the transcription factor Nkx2.1 (Figure 1A). To test this we performed *ex utero* electroporation on brain hemispheres of E14.5 mouse embryos using a mixture of pLhx6-EGFP and alexa555-conjugated control siRNA that was injected into the MGE as well as the cortex before electroporation. Alexa555 of the siRNA was used to mark the tissue where plasmid solution was injected. After preparation of slices and culturing for 1 day *in vitro* EGFP was only expressed in cells of the MGE, but not in the cortex (Figure 1B). Thus, co-transfection of pLhx6-EGFP with control or DISC1 miRNA encoding constructs (Steinecke et al., 2012) in a 1:4 ratio was performed at E13.5 to study exclusively the migration of cells transfected within the MGE. The overrun of miRNA constructs prevented false-positive cells which were only transfected with the marker construct. After 3 days *in utero* the brains were fixed and a dense focus of EGFP+ cells was visible in the MGE, making it difficult to determine the precise number of these neurons. We therefore counted the neurons that have migrated from the MGE into the LGE and compared this with the number of neurons that have migrated further and reached the cortex. Under control conditions, the number of cells in the LGE and in the cortex was roughly the same (cortex: 54.46 ± 2.10%, LGE: 45.54 ± 2.10%, 60 slices from 12 brains; Figures 1C,C′,D). In contrast, expression of DISC1 miRNA caused a significant reduction of labeled cells that arrived in the cortex. Merely 24% of the cells reached the cortex at this time point, most of the cells were stuck in the LGE (cortex: 23.99 ± 3.44%, LGE: 76.01 ± 3.44%, 17 slices from 5 brains, *p* < 0.001; Figures 1C,C′,D).

The truncated form of DISC1 that was found in a Scottish pedigree has been described as dominant negative (Kamiya et al., 2005). Transfecting MGE cells with expression vectors for shortened murine DISC1 led to a significant decrease in the number of cells in the cortex after 3 days *in utero* (cortex: 34.55 ± 2.58%, LGE: 65.45 ± 2.58%, 25 slices from 7 brains, *p* < 0.001 compared to control; Figure 1D). However, with 35% of cells reaching the cortex, truncated DISC1 caused a weaker migration defect compared to the DISC1 knockdown by miRNA.

**Figure 2:**
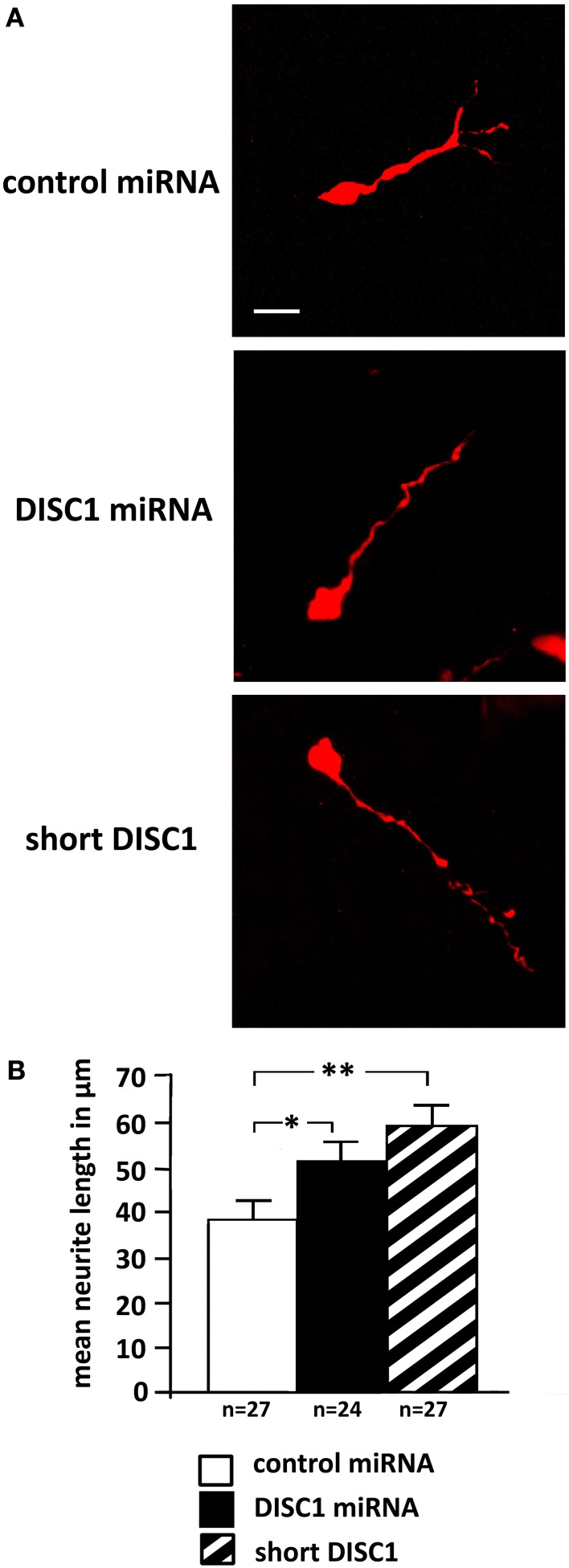
**Knockdown of DISC1 leads to prolonged leading processes *in vivo*. (A)** Photomicrographs of cells after *in utero* electroporation show alterations in process morpholgy. **(B)** Quantification of the neurite length revealed prolonged leading processes after expression of DISC1 miRNA and short DISC1. Student's *t*-test: ^*^p < 0.05, ^**^p < 0.005. Error bars: s.e.m. “n” reflects number of cells.

As our previous results showed alterations in the cell morphology after DISC1 miRNA expression (Steinecke et al., 2012), we analyzed the processes of transfected cells that had left the MGE and reached the LGE after 3 days *in vivo*. In accordance with this earlier study, cells that expressed DISC1 miRNA or a short form of DISC1 exhibited prolonged leading processes compared to control transfected cells (control: 38 ± 4 μm, 27 cells; DISC1 miRNA: 51 ± 5 μm, 24 cells, *p* < 0.05; short DISC1: 59 ± 5 μm, 27 cells, *p* < 0.01; Figures 2A,B).

Thus, using a reporter construct for Lhx6 positive cells we could demonstrate that DISC1 is an essential component for the correct migration of cortical interneurons in the intact brain. Knockdown of DISC1 as well as the expression of a dominant negative form led to a delayed migration after 3 days *in vivo*. Considering that for technical reasons we could only analyze cells that were able to leave the MGE after transfection, the migration abnormalities caused by DISC1 deficiency might be even more pronounced than described here.

### Dynamics of soma translocations after DISC1 knockdown in cortical interneurons

**Figure 3:**
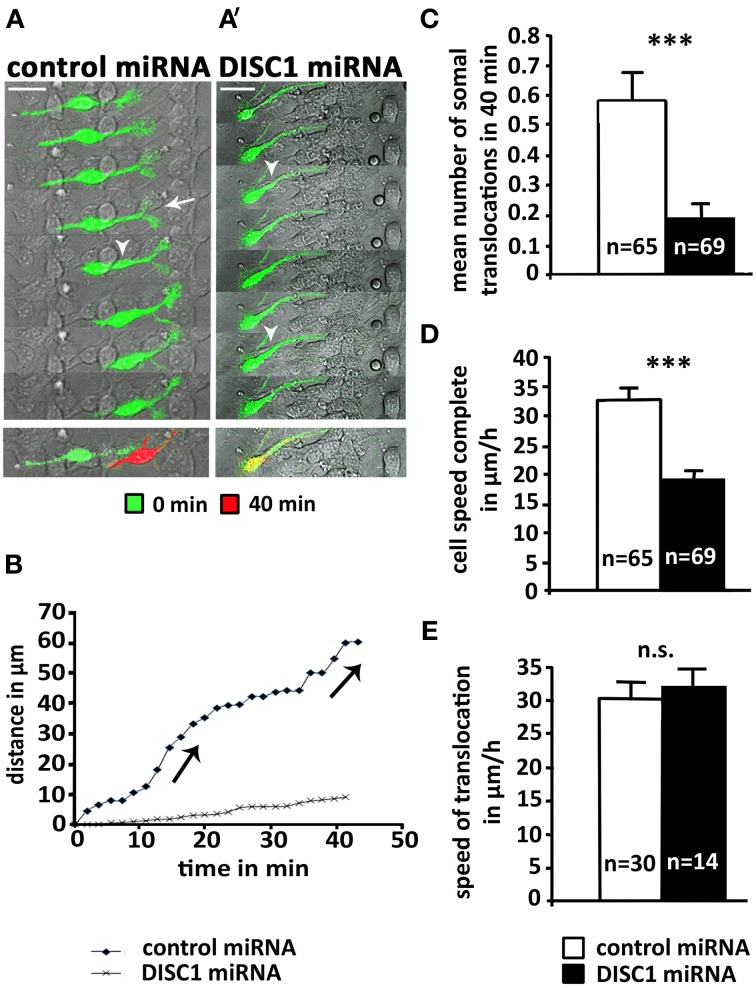
**DISC1 deficient cells perform less somal translocations *in vitro*. (A, A′)** Time line of interneurons expressing control miRNA **(A)** or DISC1 miRNA **(A′)** on a cortical feeder-layer. Arrow heads indicate swellings, arrows indicate newly built bifurcations. Time interval 10.5 min. Overlays of the time points 0 min (green) and 40 min (red) demonstrate the movement of the cells. Scale bars: 20 μm. **(B)** Graphic representation of the movement of cells mapped under **(A,A′)**. Jumps in the graph indicate somal translocations. In contrast to the control, the DISC1 deficient cells performed no somal translocations within 40 min. **(C)** Analysis of somal translocations per cell in 40 min resulted in a decreased number of translocation in DISC1 deficient cells compared to control cells. **(D)** Analysis of the migration speed in 40 min shows a reduction after DISC1 miRNA expression. **(E)** Analysis of soma translocation velocity revealed no difference. Error bars: s.e.m. Student's *t*-test: n.s. *p* ≥ 0.05, ^***^p < 0.001, “n” reflects number of cells.

To understand the underlying cellular mechanism of how DISC1 regulates interneuron migration we went back from the *in vivo* approach to *in vitro* systems. Therefore, we injected plasmid solution into the MGE of preparated hemisphäres and electroporated them *ex utero*. Afterwards single cells were generated and cultured on cortical feeder-layers. Using time-lapse video microscopy to visualize the complex migration behavior of interneurons it was possible to analyze the dynamic of individual migrating cells on a two-dimensional substrate. Figure 3A depicts a typical cell transfected with control miRNA. As illustrated in Figure 3B, two soma translocations occurred within the 40 min recording period. In addition, a highly dynamic growth cone-like structure is visible at the leading process tip exhibiting bifurcations (arrow) and swellings (arrow head). In contrast, the DISC1 miRNA transfected cell in Figure 3A′ exhibited a thin, prolonged and less branched leading process and the growth cone-like structure was greatly reduced compared to control cells. Although swellings (arrow heads) appeared several times, a soma translocation did not occur. A graphic representation of the trajectories of these two cells is illustrated in Figure 3B. Whereas the control transfected cell exhibits the typical saltatory pattern of interneuron migration, with leading process extension followed by soma translocation and retraction of the trailing process, there was almost no translocation of the DISC1 deficient interneuron, while the leading process was constantly moving. Consistently, in contrast to the control, no trailing process was identifiable in these cells (Figure 3A′).

Analyzing all cells monitored within 40 min we found a significant reduction of soma translocations after DISC1 knockdown. About half of the control cells performed at least one soma translocation within the 40 min recording period (30 out of 65), whereas only 14 out of 69 DISC1 deficient neurons exhibited a soma translocation. The mean number of soma translocations was 0.6 ± 0.1 after transfection with control miRNA and 0.2 ± 0.1 after transfection with DISC1 miRNA (*p* < 0.001; Figure 3C). In accordance with this observation, the mean velocity of DISC1 deficient interneurons (19 ± 1 μm/h) was significantly lower than for control neurons (33 ± 2 μm/h; *p* < 0.001, Figure 3D). However, when translocations occurred in DISC1 deficient interneurons, this arose at the same speed as in control cells (control miRNA: 30 ± 2 μm/h, 30 cells; DISC1 miRNA: 32 ± 2 μm/h, 14 cells; *p* > 0.05, n.s.; Figure 3E).

In conclusion, after DISC1 knockdown MGE-derived interneurons display less soma translocations on a cortical feeder-layer than control cells. The velocity of soma translocation is not altered in DISC1 deficient interneurons, suggesting that their migration defects are caused by alterations of the intracellular coordination between several components of the migration machinery.

### Dynamics of the actin cytoskeleton after DISC1 knockdown in cortical interneurons

**Figure 4:**
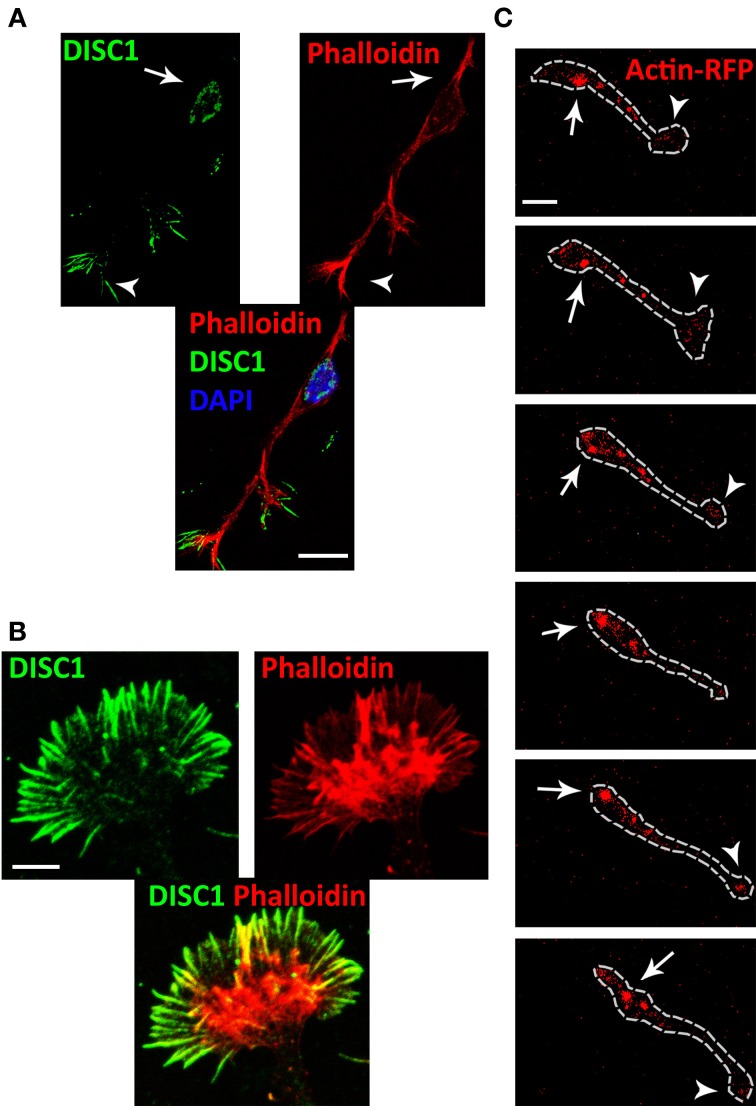
**DISC1 co-localizes with F-actin in migrating interneurons. (A)** Fluorescent micrographs of interneurons stained with phalloidin and α-DISC1 antibodies. Arrows indicate an expression of DISC1 or F-actin behind the nucleus, arrowheads within the leading process tip. Scale bar: 10 μm. **(B)** High magnification of a growth cone-like structure reveals the co-localization of DISC1 and F-actin. Scale bar: 5 μm. **(C)** Time line of an actin-RFP expressing interneuron migrating on a cortical feeder-layer. Actin-RFP shows a consistent condensation in the leading process tip (arrowheads) and a dynamic condensation in the soma (arrows). During soma translocation actin-RFP spots move from the initial segment of the leading process to the cell rear. Scale bar: 10 μm.

Previous studies provided evidence that the actin cytoskeleton is essential for glia-independent migration of neurons, exerting pushing and/or pulling forces (He et al., 2010; Martini and Valdeolmillos, 2010). Therefore, we examined the distribution of actin and DISC1 at the subcellular level in cortical interneurons. For this phalloidin staining of interneurons migrating in a 3D substrate of plasma was used to label filamentous (F)-actin in combination with DISC1 immunostainings using an antibody raised against a peptide mapping near the N-terminus of the DISC1 protein. Confirming our previous results (Steinecke et al., 2012), both DISC1 and F-actin were located behind the nucleus and in the tips of the leading processes (Figure 4A). Within the growth cone-like structure, DISC1 is in front of F-actin in the filiopodia, as illustrated at high magnification in Figure 4B.

To monitor actin dynamics in migrating interneurons we performed time-lapse video microscopy of actin-RFP expressing MGE cells on a cortical feeder layer. In this experiment, the fluorescence RFP signal reports both globular (G-) actin as well as F-actin. However, counterstaining of transfected cells with phalloidin indicated that highly condensed RFP indicates predominantly F-actin (Lee et al., 2013). Our time-lapse movies reveal that during migration on cortical feeder-layers dynamic condensations of actin filaments occurred within the cell soma as well as in the leading process tip of interneurons. F-actin signals appeared in front of the nucleus and moved sidewise to the back during soma translocation (Figure 4C and Supplementary Movie). Within the highly dynamic growth cone-like structure accumulation of actin-RFP occurred frequently.

**Figure 5:**
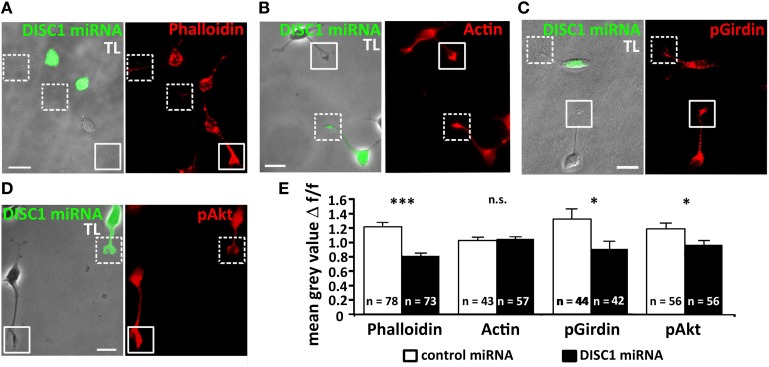
**Knockdown of DISC1 affects the actin cytoskeleton in migrating interneurons. (A–D)** Fluorescence micrographs of DISC1 miRNA expressing (green) and untransfected cells stained with phalloidin **(A)**, α-actin **(B)**, α-pGirdin **(C)**, and α-pAkt **(D)** antibodies. Frames display the tips of the leading processes. TL, transmitted light. Scale bars: 10 μm. **(E)** Quantification of the fluorescence intensities of control miRNA as well as DISC1 miRNA expressing cells. The ratio of the mean gray value (relative fluorescence intensity) of transfected and non-transfected cells was calculated. In their leading process tips DISC1 deficient cells displayed a weaker phalloidin, pAkt, and pGirdin, but not actin signal. Student's *t*-test: n.s. *p* ≥ 0.05, ^*^p < 0.05, ^***^p < 0.001. Error bars: s.e.m. “n” reflects number of cells.

The growth cone-like structure of interneurons is more elaborated than in radial migrating cortical projection neurons (Rakic, 1971; Bellion et al., 2005). It has been demonstrated that severing the tip of the leading process or restricting its dynamics stopped soma translocation (He et al., 2010). To study if DISC1 knockdown induced alterations of the actin cytoskeleton we analyzed the growth cone-like structure of fixed control miRNA and DISC1 miRNA expressing interneurons and compared their relative fluorescence intensities. In DISC1 deficient cells phalloidin staining was considerably less intense than in control miRNA transfected cells (control miRNA/untransfected: 1.21 ± 0.07, 78 cells; DISC1 miRNA/untransfected: 0.81 ± 0.04, 73 cells; *p* < 0.001; Figures 5A,E) indicating a decrease of F-actin in the leading process tips. To examine whether this reduction of filaments resulted from a decline in protein levels we performed immunocytochemistry using a pan antibody, binding both to G- and F-actin. There was no difference in the fluorescence intensities between cells expressing control or DISC1 miRNA (control miRNA/untransfected: 1.03 ± 0.05, 43 cells; DISC1 miRNA/untransfected: 1.05 ± 0.04, 57 cells; n.s. *p* > 0.05, n.s.; Figures 5B,E).

Previous studies indicated that Girdin (girders of actin filaments) is one of the many interaction partners of DISC1, which cross-links actin filaments (Enomoto et al., 2006, 2009). Moreover, it has also been demonstrated that Girdin is a regulator of neuroblast chain migration in the rostral migratory stream of the postnatal brain and the number of cortical interneurons is significantly decreased in Girdin^−/−^ mice compared with wild-type animals (Wang et al., 2011). We therefore analyzed the amount of activated, phosphorylated Girdin (pGirdin) in leading process tips of cortical interneurons. After DISC1 knockdown the pGirdin signal was reduced by 30% compared to control levels (control miRNA/untransfected: 1.32 ± 0.97, 44 cells; DISC1 miRNA/untransfected: 0.91 ± 0.70, 42 cells; ^*^p < 0.05; Figures 5C,E).

Although DISC1 was discovered as a binding partner of Girdin, so far there is no evidence that DISC1 directly activates Girdin. However, one known activator of Girdin is Akt, also known as protein kinase B (PKB), a serine/threonine kinase. Activated Akt (pAkt) phosphorylates serine at position 1416 in Girdin, and activated pGirdin accumulates at the leading edge of migrating cells (Enomoto et al., 2005, 2009). Therefore, we examined the effect of DISC1 knockdown on pAkt levels in the growth cone-like structure of migrating cortical interneurons and found a significant reduction compared to control cells (control miRNA/untransfected: 1.19 ± 0.08, 56 cells; DISC1 miRNA/untransfected: 0.97 ± 0.07, 56 cells; *p* < 0.05; Figures 5D,E).

Taken together, these results indicate that leading process tips of DISC1 deficient interneurons display lower F-actin levels compared to control. The decrease of activated forms of Girdin and Akt, which are responsible for the cross-linking of actin filaments, supports the idea that DISC1 knockdown influences the actin cytoskeleton in the growth cone-like structures of migrating interneurons and thereby interferes with their migration.

### Alterations of microtubules of DISC1 deficient cortical interneurons

**Figure 6:**
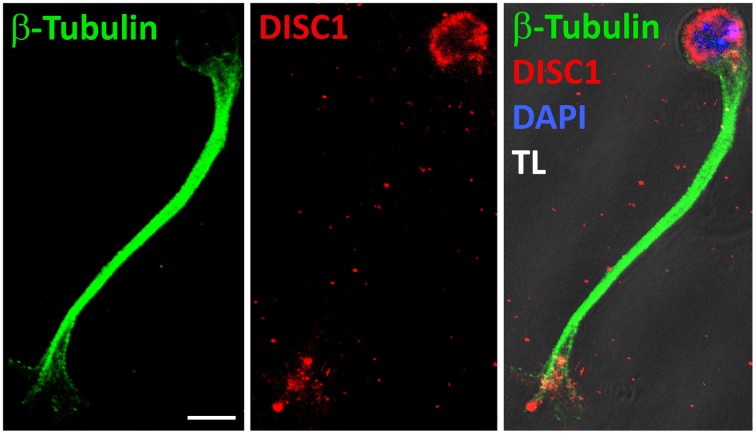
**DISC1 overlaps with microtubules in the growth cone-like structure**. Fluorescent micrographs of a migrating interneuron stained with α-Tubulin as well as α-DISC1 antibodies. The leading process shaft is free of DISC1, but filled with tubulin fibers. TL, transmitted light. Scale bar: 5 μm.

So far we analyzed the effects of a DISC1 knockdown on the actin cytoskeleton in the leading process tip. But the growth cone-like structure is unlikely characterized by actin only. In axonal growth cones different forms of actin are functionally linked to microtubules which also play an essential role during growth cone dynamics. Immunostaining of MGE cells migrating in a 3D substrate revealed that the leading process shaft is completely filled with microtubules which partially enter the growth cone-like structure where DISC1 is expressed (Figure 6, Steinecke et al., 2012). This as well as the observed alterations of the leading process morphology (Figure 2, Steinecke et al., 2012) imply effects on the microtubule cytoskeleton after DISC1 knockdown. We therefore analyzed the microtubule cytoskeleton in DISC1 deficient cells.

**Figure 7:**
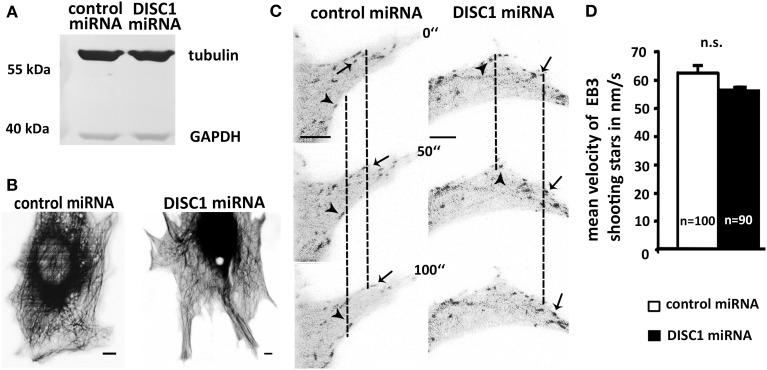
**DISC1 deficient cells exhibit neither alterations in microtubule polymerization nor integrity. (A)** Western blot analysis of control miRNA and DISC1 miRNA expressing fibroblasts revealed no change in the tubulin level after DISC1 knockdown. **(B)** Inverted fluorescent micrographs of NIH3T3 fibroblasts expressing control or DISC1 miRNA as well as Tubulin-GFP. Scale bar: 5 μm. **(C)** Time line of NIH3T3 fibroblasts that expressed control or DISC1 miRNA as well as EB3-GFP. Arrowheads and arrows display EB3-GFP proteins (“shooting stars”) that were followed over time. **(D)** Analysis of the EB3 shooting star velocity showed no difference between control and DISC1 miRNA expressing NIH3T3 fibroblasts. Student's *t*-test: n.s. *p* ≥ 0.05. Error bars: s.e.m. “n” reflects number of shooting stars.

First, we examined the overall tubulin level of NIH3T3 that were transfected with control or DISC1 miRNA encoding vectors using western blot analysis. Reduction of DISC1 led neither to an increase nor a decrease of tubulin on the protein level in those cells (Figure 7A).

As the tubulin level seemed not to be changed, we investigated the effect of the DISC1 knockdown on microtubule polymerization and network integrity. For this we visualized the microtubule cytoskeleton in NIH3T3 fibroblasts and performed time lapse video microscopy. Using fluorescence-tagged tubulin we were able to monitor the whole microtubule cytoskeleton of living miRNA expressing fibroblasts. Like under control conditions DISC1 deficient cells displayed a compact and shape filling tubulin network involving elongated and curled regions with stable as well as dynamic microtubules. Conspicuities like microtubules that did not reach the cell cortex or instable microtubules that were consistently retracted could not be observed (Figure 7B).

Next we analyzed the velocity of tubulin polymerization using GFP-tagged EB3 (end-binding protein 3). This microtubule binding protein is specifically associated with the ends of growing microtubules and therefore enables the visualization of microtubule growth in living cells (Stepanova et al., 2003). Analyzing these EB3-GFP signals that appear over time (“shooting stars”) we found no difference between control and DISC1 miRNA expressing NIH3T3 fibroblasts which means neither an increased nor a reduced velocity of microtubule polymerization after DISC1 knockdown (Figures 7C,D; control miRNA: 62.5 ± 2.8 nm/s, 100 shooting stars, 14 cells; DISC1 miRNA: 56.8 ± 4.1 nm/s, 90 shooting stars, 15 cells; n.s. *p* > 0.05).

**Figure 8:**
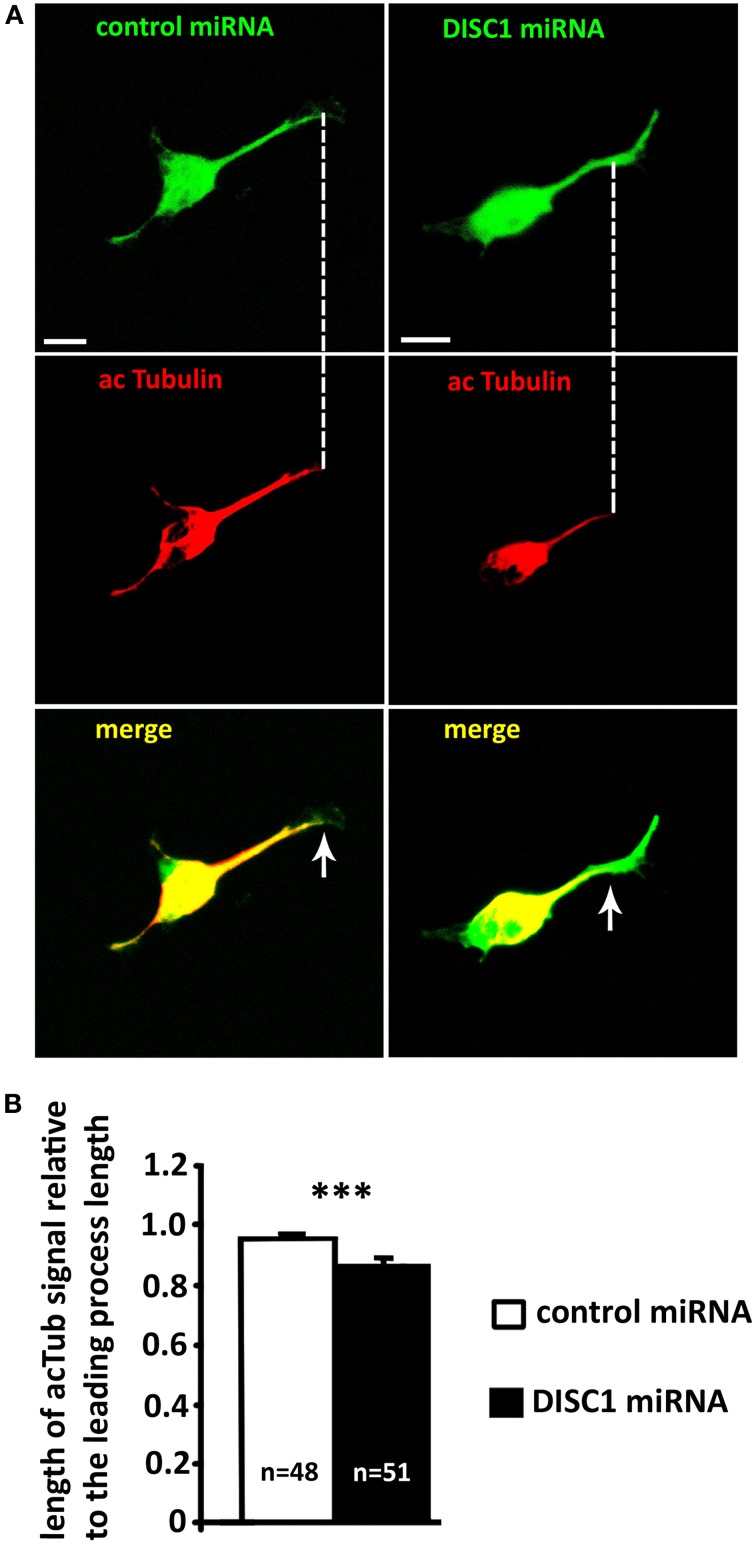
**Knockdown of DISC1 affects microtubule acetylation in the leading process of migrating interneurons. (A)** Fluorescence micrographs of control and DISC1 miRNA expressing interneurons immunostained against acetylated Tubulin (acTub). Arrows display the end of the acTub expression in the leading processes. Scale bars: 5 μm. **(B)** Analysis of the acTub signal in the leading process relative to the leading process length. DISC1 knockdown resulted in a decreased length of acTub compared to control cells. Student's *t*-test: ^***^p < 0.001. Error bars: s.e.m. “n” reflects number of cells.

In growing axons the stabilization of microtubules plays an essential role in the correct movement of the growth cone. Stable bundles of microtubules fill the axon shaft and extend into the growth cone. There they are belt by actin arcs and stabilized by microtubule-associated proteins. Single dynamic filaments reach out of the stable bundles and explore the periphery. Since dynamic microtubules act as guidance sensors, stable bundles are necessary for axonal forward movement. Locally induced stabilization and dynamic are required for growth cone turning and branching. (reviewed in Lowery and Van Vactor, 2009; Kalil and Dent, 2014). To examine whether a DISC1 knockdown effects the selective stabilization of microtubules in the growth cone-like structure of migrating interneurons, we analyzed the acetylation of microtubules in fixed MGE cells. Although this post-translational modification seems not the have an influence on polymerization or depolymerization of microtubules by itself, it is presumed to be a marker for stabilization (Schulze et al., 1987; Westermann and Weber, 2003). We performed immunocytochemistry on MGE cells and found a significant decrease of acetylation at the distal end of the leading process after DISC1 knockdown. In control cells from the cell body into the growth cone-like structure. In DISC1 deficient cells only 87% of the leading process length from the cell body toward the leading process tip was acetylated (Figures 8A,B; control miRNA: length of acetylated tubulin signal relative to the leading process length: 0.96 ± 0.01, 48 cells; DISC1 miRNA: length of acetylated tubulin signal relative to the leading process length: 0.87 ± 0.02, 52 cells; ^***^p < 0.001). Thus, stable microtubule bundles are no longer reaching into the growth cone-like structure in DISC1 deficient cortical interneurons.

Taken together, in interneurons with decreased DISC1 levels microtubule stabilization is reduced at the leading process tip where stable microtubule bundles reach into the growth cone-like structure. This effect seems not to be mediated by altered polymerization and dynamic of microtubules.

## Discussion

More than a decade after the first description of DISC1 as a susceptibility gene for major psychiatric disorders, multiple studies attempted to trace its potential functions during brain development. It has become clear that DISC1 is crucial for the correct development of several telencephalic structures by regulation, e.g., cell division, synapse formation or neuronal migration (reviewed in Brandon et al., 2009; Soares et al., 2011; Narayan et al., 2013). Using a variety of *in vitro* assays, in our previous study we found evidence that DISC1 knockdown by RNA intereference results in a migration defect of MGE-derived cortical interneurons (Steinecke et al., 2012). Here we first demonstrate that DISC1 is essential for the proper tangential migration of cortical interneurons *in vivo*. We then examined that underlying cellular and molecular mechanisms that lead to the migration defects of DISC1 deficent interneurons. Our results indicate that DISC1, via interactions with Akt and Girdin, influences network structure and stabilization of actin filaments. In addition, DISC1 also influences the stability of microtubules but had no effect microtubule polymerization.

### DISC1 plays a critical role in tangential migration of cortical interneurons

Although some DISC1 models have been generated (Cash-Padgett and Jaaro-Peled, 2013), up to now there is no DISC1 knockout mouse available to study directly the potential of DISC1 during brain development. Therefore, we used RNA interference techniques to study the impact of DISC1 on neuronal migration. By performing *in utero* electroporation we were able to reduce the level of DISC1 in cells of the ganglionic eminences at a defined time point during development. It has been claimed that it is possible to aim exclusively the MGE with this technique (Borrell et al., 2005). However, we and others were not able to restrict the transfection region to this part of the eminences, but rather almost always got many labeled cells in the basal as well as in the dorsal telencephalon after electroporation (Bai et al., 2008; Steinecke et al., 2012). To overcome this problem and to analyze only MGE-derived cells we used a marker that is expressed exclusively in Lhx6^+^ cells, a transcription factor specific for postmitotic cortical interneurons generated in the MGE. This was confirmed by injecting plasmids in the MGE and cortex in brain slices. After electroporation, labeled neurons were only found in the MGE and not in the dorsal telencephalon. Using this approach, we found that after DISC1 knockdown fewer interneurons reached the cortex compared to control transfected cells. Similar experiments with a dominant-negative form of DISC1 also lead to migration defects, although they have been less pronounced compared to DISC1 knockdown.

Previous studies found abnormal migration after DISC1 knockdown in cortical projection neurons which migrate radially from the ventricular zone into the developing cortical plate (Kamiya et al., 2005; Kubo et al., 2010; Young-Pearse et al., 2010). Delayed migration has also been described for DISC1 deficient neurons in the developing hippocampus (Meyer and Morris, 2009; Tomita et al., 2011). Surprisingly, in the adult hippocampus, newly generated neurons over-migrated their target after DISC1 knockdown (Duan et al., 2007; Enomoto et al., 2009; Kim et al., 2009). Thus, DISC1 seems to have opposite effects on neuronal migration of developing and adult hippocampal neurons. Finally, DISC1 seems to have no impact on postnatal neuroblast migration within the rostral migratory stream (Wang et al., 2011). Thus, the influence of DISC1 on neuronal migration appears to be cell type specific and can also depend on the developmental stage of the neurons.

The loss of DISC1 binding partners can also lead to defects in neuronal migration. A prominent example is LIS1 that interacts with DISC1 at the centrosome (Kamiya et al., 2005). LIS1 deficient cortical projection neurons as well as cortical interneurons exhibit a delayed migration. This is caused by the reduction of the rate of nuclear movement, whereas process extension remains unaffected, resulting in prolonged and less branched leading processes (McManus et al., 2004; Nasrallah et al., 2006; Gopal et al., 2010). These results are reminescent to effects observed after DISC1 knockdown (Steinecke et al., 2012, present results). The fact that impairment of LIS1 function results in migration defects and causes lissencephaly (Kato and Dobyns, 2003) underlines the relevance of DISC1 and its interacting partners during brain development.

### DISC1 interferes with the actin cytoskeleton in migrating interneurons

Tangential migration of cortical interneurons differs from radial migration in several respects (reviewed in Nadarajah and Parnavelas, 2002; Marin and Rubenstein, 2003). Unlike cortical projection neurons, cortical interneurons originate in the ventral telencephalon and therefore have to migrate over much longer distances to reach their final destination in the developing cortex. As these cells have to find their way without guidance of glial fibers they exhibit a distinctive growth cone-like structure at the leading process tip which is thought to “explore” the environment and make steering decisions. (reviewed in Marin and Rubenstein, 2001). Its branched and highly dynamic appearance is crucial for interneuron migration (Bellion et al., 2005; Metin et al., 2006; Valiente and Martini, 2009; He et al., 2010). During tangential migration cortical interneurons consistently repeat two steps: (I) elongation of the leading process and (II) soma translocation combined with elongation stop and branching of the growth cone-like structure (Bellion et al., 2005). Accordingly, suppression of soma translocation by blocking the actin-associated motor protein nonmuscle myosin II or inhibition of ROCK, a Rho effector that regulates myosin II activity results in prolonged and very thin processes (Bellion et al., 2005; Shinohara et al., 2012). Likewise, in this study knockdown of DISC1 decreased the number of soma translocations on cortical feeder-layers and caused the elongation of leading processes.

Several previous studies indicate a crucial role of actin dynamics during soma translocation. It has been shown that nucleokinesis is associated with a precise pattern of actin condensations characterized by the initial formation of a cup-like structure at the rear nuclear pole. This is followed by a progressive actomyosin contraction that drives the nucleus forward and concludes with an actin spot at the base of the retracting trailing process (Bellion et al., 2005; Martini and Valdeolmillos, 2010; Shinohara et al., 2012). Furthermore, enriched F-actin signals have been observed in the leading process tip and also in front of the nucleus where they appear prior to translocation events and move to the direction of the leading process (Shinohara et al., 2012). It has been proposed that the soma is actively pulled forward by a F-actin flow along the leading process. This is based on results from migrating cerebellar granule cells where somal translocation is suppressed by severing the leading process tip and/or local disruption of F-actin along the leading process. Experiments using localized application of F-actin stabilizing and destabilizing agents revealed the necessity of actin dynamics especially within the growth cone-like structure at the leading process tip. In contrast, local perfusion of actin modulating agents at the cell soma has no effect on nucleokinesis (He et al., 2010). Using time-lapse video microscopy of actin-RFP expressing MGE cells we found evidence for both models of pushing and pulling the nucleus forward (Figure 4C).

Given that actin remodeling is necessary for the correct interneuron migration we investigated the role of DISC1 on the actin cytoskeleton. For this we analyzed the leading process tips after DISC1 knockdown and found less F-actin in the growth cone-like structure compared to control cells. This result is comparable to the effect of a LIS1 knockdown in radially migrating neurons, which also display a migration defect as described above (Kholmanskikh et al., 2003). Thus, DISC1 seems to have an impact on F-actin remodeling at the leading edge, which is essential for neuronal migration.

A binding partner of DISC1 involved in actin remodeling is Girdin (Camargo et al., 2007). After phosphorylation by Akt it detaches from the cell membrane and cross-links newly built actin filaments (Enomoto et al., 2006). Currently, there are two different hypothesis about the role of DISC1 and Girdin in newborn granule cells of the adult hippocampus. According to Kim et al. (2009), DISC1 prevents the interaction of Girdin and Akt and therefore decreases the activation of Girdin by Akt. In contrast, another study points out the function of DISC1 to localize Girdin in growth cones thereby promoting Girdin activation at the axonal tip (Enomoto et al., 2009). Here we show that knockdown of DISC1 caused a reduction of F-actin, activated Girdin as well as activated Akt in the growth cone-like structure of cortical interneurons. Although it is not clear how DISC1 and Girdin interact, these results indicate a role of DISC1 in actin remodeling during tangential migration.

**Figure 9:**
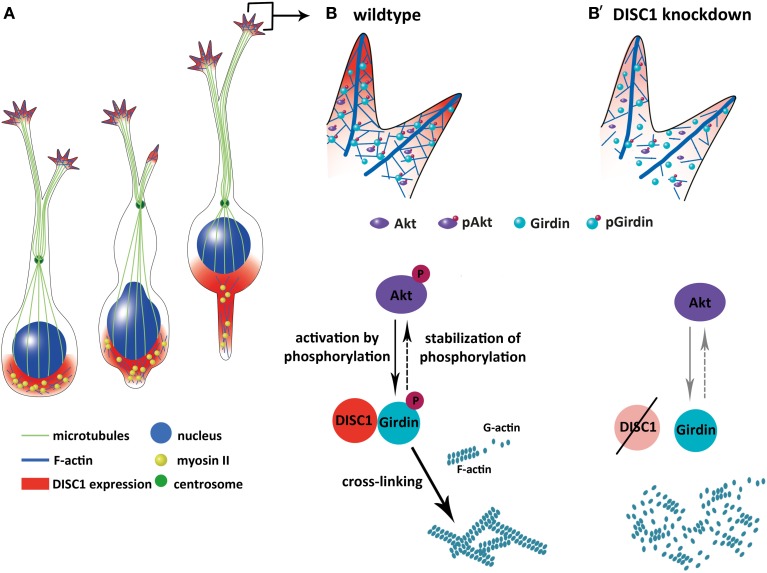
**Model how DISC1 orchestrates the tangential migration of cortical interneurons. (A)** Soma translocation during tangential migration. DISC1 co-localizes with F-actin behind the nucleus and in the growth cone-like structure, where actin forces drive soma translocation. **(B,B′)** Interaction of DISC1 with Girdin provides a possible link between DISC1 and actin reorganization which is necessary for correct soma translocations. In wildtype interneurons **(B)**, phosphorylated forms of Akt and Girdin lead to cross-linked F-actin. In the absence of DISC1 **(B′)**, the amount of phosphorylated Akt as well as Girdin is decreased, an effect of DISC1 on the activation of these proteins. However, according to Kim et al. (2009) it is also possible that DISC1 is necessary for the proper localization of Girdin in the growth cone-like structure of migrating cortical interneurons. According to this scenario, a loss of DISC1 would also cause a reduction of non-phosphorylated Girdin. Although it is not completely clear how the three proteins DISC1, Akt and Girdin influence each other, reduced F-actin and increased G-actin levels indicate an impact of DISC1 on actin remodeling events resulting in less stabilized actin filaments.

In summary, DISC1 is expressed in the leading process tip as well as in the cell soma mainly behind the nucleus of migrating MGE cells (Figure 9, Steinecke et al., 2012) overlapping with phalloidin stained F-actin. Considering the growth cone-like structure at the end of the leading process reduced expression levels of DISC1 lead to less F-actin since the over all protein level of actin is not decreased. Additionally, reduction of active forms of the actin cross-linking protein Girdin and its activator Akt further confirm the disruption of actin remodeling caused by DISC1 deficiency. This points to the hypothesis that DISC1 knockdown impairs the actin cytoskeleton in tangentially migrating cortical interneurons, finally resulting in a restricted ability to perform soma translocations, as depicted in Figure 9.

### Knockdown of DISC1 interferes with microtubule stabilization in leading process tips of migrating interneurons

In addition to actin, the microtubule cytoskeleton plays an essential role during brain development. For glia-guided migrating cells it has been shown that microtubule organization is not solely responsible for the polarity, but also participates in migration. The centrosome as the main microtubule organization center is located in front of the nucleus. The correct localization of centrosome-associated proteins ensures its functionality and is necessary for the accurate radial migration of cortical neurons in the neocortex (Higginbotham and Gleeson, 2007). Microtubules spreading out of the centrosome built a cup-like structure surrounding the nucleus (Rivas and Hatten, 1995; Xie et al., 2003; Tsai et al., 2007) and reach into the leading process where they are linked to the cellular cortex (Asada and Sanada, 2010). It has been suggested that the DISC1 binding partner LIS1 activates the dynein motor complex, which is necessary for the coupling of the centrosome and the nucleus. During somal translocation the motor complex moves along microtubule fibers and pulls the nucleus forward. Loss of function of dynein and other components of the motor complex like LIS1 causes uncoupling of the centrosome from the nucleus and impairs neuronal migration and positioning (Shu et al., 2004).

In contrast, several studies found no impact of microtubule dynamics on nucleokinesis during tangential migration of cortical interneurons (He et al., 2010; Martini and Valdeolmillos, 2010). Thus, the microtubule network is necessary basically to build the leading process and to act as a guide for the nucleus. However, also in tangentially migrating interneurons microtubule-dependent pulling forces in addition to actomyosin-dependent pushing forces have also been considered (Bellion et al., 2005). Thus, pulling as well as pushing mechanisms are possible in all migrating neurons whereupon the substrate that has a considerable impact on adhesion, is also crucial for the locomotion of cells (Bellion et al., 2005; He et al., 2010; Martini and Valdeolmillos, 2010; Luccardini et al., 2013). However, a recent study indicates an important role of microtubule dynamics in migrating MGE-derived cells. Prior nucleokinesis the centrosome moves forward along extracentrosomal microtubules into the leading process where it docks to the cellular membrane and exposes a primary cilium at the cell surface. Nuclear translocation occurs along microtubule bundles comprising extracentrosomal microtubules followed by detaching of the centrosome from the cellular membrane suggesting involvement of the microtubule cytoskeleton in nucleokinesis too (Baudoin et al., 2012).

DISC1 has been described previously to associate with microtubular organization and dynamics. Like LIS1 it is a part of the dynein motor complex and accumulates as well as stabilizes other centrosomal proteins at the centrosome and therefore up-regulates its function. Accordingly, overexpression of LIS1 as well as DISC1 leads to microtubule accumulation in the cell periphery and knockdown to a disorganized network in COS-7 cells (Smith et al., 2000; Kamiya et al., 2005). Additionally to its role in dynein-based microtubule-associated transport, DISC1 modulates the transport of organelles and components of the cytoskeleton through its direct interaction with kinesin-1 (Taya et al., 2007; Wang and Brandon, 2011). However, analyzing assembly and integrity of the microtubule cytoskeleton in DISC1 deficient NIH3T3 fibroblasts we could not observe any impact of DISC1 on microtubule organization. Cells with reduced expression levels of DISC1 displayed the same microtubule behavior and EB3 dynamics as control cells indicating no effect on polymerization and depolymerization of microtubule fibers.

In axonal growth cones, branch formation is initiated by actin and then followed by microtubule invasion. *S*tabilized microtubule bundles extent into the growth cone where they interact with actin arcs and actin filaments. Explorative dynamic microtubule fibers reach into the periphery and respond to guidance cues from the environment. Since complete inhibition of microtubule dynamics prevents turning of the growth cone, localized stabilization induces turning (reviewed in Lowery and Van Vactor, 2009; Kalil and Dent, 2014). To identify possible effects of a DISC1 knockdown on microtubule-based functions of the growth cone-like structure we analyzed microtubule stabilization in leading processes of MGE cells. Reduced stabilization at the distal ends of the processes indicates an effect on the functionality of the steering apparatus as stabilized microtubule bundles do not reach into the growth cone-like structure. This was also observed in LIS1 deficient interneurons, further confirming the close relationship between these two proteins (Gopal et al., 2010). How this effect on the microtubule cytoskeleton is mediated and whether it also has an impact on the integration of guidance cues has to be examined in future studies.

### Conflict of interest statement

The authors declare that the research was conducted in the absence of any commercial or financial relationships that could be construed as a potential conflict of interest.
